# A phase I trial of azacitidine and nanoparticle albumin bound paclitaxel in patients with advanced or metastatic solid tumors

**DOI:** 10.18632/oncotarget.14183

**Published:** 2016-12-26

**Authors:** Adam L. Cohen, Abhijit Ray, Matthew Van Brocklin, David M. Burnett, Randy C. Bowen, Donna L. Dyess, Thomas W. Butler, Theresa Dumlao, Hung T. Khong

**Affiliations:** ^1^ Department of Medicine, Division of Oncology, University of Utah, Huntsman Cancer Institute, Salt Lake City, UT, USA; ^2^ Division of Oncology, University of Utah, Huntsman Cancer Institute, Salt Lake City, UT, USA; ^3^ Department of Surgery, University of Utah, Huntsman Cancer Institute, Salt Lake City, UT, USA; ^4^ Department of Medicine, University of Utah, Salt Lake City, UT, USA; ^5^ University of South Alabama, Mitchell Cancer Institute, Mobile, AL, USA; ^6^ Banner MD Anderson Cancer Center, Gilbert, AZ, USA

**Keywords:** nab-paclitaxel, azacitidine, solid tumor, phase I clinical trials, SPARC

## Abstract

**Background:**

Secreted protein acidic and rich in cysteine (SPARC), an albumin-binding protein, is downregulated by hypermethylation in many cancers. Hypomethylating agents such as azacitidine can upregulate SPARC in tumors, which may enhance the accumulation of albumin-bound drugs at tumor site. The objectives of this phase I trial was to determine the safety and maximum tolerated dose and to assess any clinical activity of the combination of azacytidine and weekly nanoparticle-albumin-bound (nab®) paclitaxel.

**Methods:**

Patients received escalating azacytidine doses daily for 5 days, followed by nab-paclitaxel at the standard 100mg/m2 weekly dose for 3 weeks in 4-week cycles. Dose-limiting toxicities (DLTs) were monitored during the first cycle. Serum was obtained at baseline, during and after treatment for correlative study.

**Results:**

All sixteen total patients enrolled were evaluable for toxicity, while 13 patients were evaluable for response. Two of five patients treated with 100mg/m2 of azacytidine had DLT of prolonged grade 4 neutropenia. Therefore, the MTD of azacitidine in this regimen is 75 mg/m^2^. Three additional patients were treated with no grade 4 toxicity in cycle 1. Clinical activity included 1 complete response (CR) in refractory DLBCL, 2 CR in ovarian cancer, 4 partial responses (PR) in ovarian and endometrial cancer, 4 stable diseases (SD) in lung, sarcoma and pancreatic cancer, 1 unconfirmed PR in breast cancer, and 1 progression of disease in CLL/SLL.

**Conclusions:**

Priming with azacitidine 75 mg/m^2^ daily for 5 days, followed by weekly nab-paclitaxel 100 mg/m^2^ weekly was well tolerated and results in dramatic responses pre-treated cancer patients.

## INTRODUCTION

Paclitaxel, a semisynthetic antineoplastic agent, is FDA approved as a single agent or in combination with other drugs and constitute some of the most active and commonly used drugs as a first line of treatment. Limitations in the use of paclitaxel is that it is highly hydrophobic requiring synthetic solvents to deliver therapeutic doses of the drug [1]. Paclitaxel requires a combination of polyethylated castor oil and ethanol (Cremophor EL) and these solvents contribute to hypersensitivity and allergic reactions requiring premedication [1]. Nanoparticle-albumin-bound (nab®) paclitaxel combines a protein with a chemotherapeutic agent in the particle form and can be delivered without the use of these synthetic solvents and is approved for the treatment of metastatic breast cancer [2], advanced non-small cell lung cancer (NSCLC) [3], and late stage pancreatic cancer [3]. Nab-paclitaxel has been studied in multiple dosing schemes. Weekly nab-pacliaxel at 100mg/m^2^ for three out of every 4 weeks is well tolerated and produces responses even in heavily pretreated women with taxane-resistant breast cancer [4]. This composition provides a novel approach for increasing intratumoral concentration of the drug by a receptor-mediated transport process allowing transcytosis across the endothelial cell wall. This process is hypothesized to involve the activation of the albumin-specific receptor gp60 on the endothelial cell wall, resulting in activation of caveolin-1, which in turn initiates an opening in the endothelial wall with formation of caveolae and transport of the albumin-bound chemotherapy complex via these caveolae to the underlying tumor interstitium [5].

A protein secreted by the tumor, secreted protein acidic and rich in cysteine (SPARC) is postulated to bind and entrap the albumin, allowing release of the hydrophobic drug to the tumor cell membrane [6]. SPARC, is a secreted glycoprotein that forms a transient component of the extracellular matrix (ECM) and is involved in morphogenesis, tissue remodeling, and cell migration and proliferation through cell-ECM interactions [7–9]. SPARC has also been found to interact with other components of the ECM and to regulate the expression and function of matrix metalloproteinase [7–9]. In some tumor types, SPARC has been shown to act as a tumor suppressor [10–12]. The underlying mechanisms for this function are not clear [13–15]. Using gene-expression microarray, SPARC has been shown to be a putative resistance-reversal gene. Re-expression of SPARC conferred radio- and chemosensitivity to resistant colon cancer cells in a xenograft mouse model [10]. Decreased SPARC expression due to promoter hypermethylation has been seen in pancreatic cancer, lung cancer, cervical cancer, and ovarian cancer [11, 12, 16–18].

Azacitidine, an analog of the pyrimidine nucleoside cytidine, inhibits DNA methylation and is approved for the treatment of myelodysplastic syndrome [19–21]. The hypomethylating agent azacytidine was able to upregulate SPARC expression in most cases [22]. We hypothesized that pretreatment with azacytidine could decrease methylation of the SPARC promoter, increase SPARC expression in tumors, and increase sensitivity to nab-paclitaxel.

The rationale of the study is that by exploiting both caveolin-1 and the SPARC protein, nab-paclitaxel may preferentially enhance drug delivery to tumors. The cytotoxicity of azacitidine is proportional to dose and exposure time (23, 24]. Therefore, we designed a phase I dose escalation trial to determine the maximum tolerated dose of azacytidine to combine with nab-paclitaxel. Finally, with the increase in the interstitial pressure inside the solid tumors, resulting in the collapse of the lymphatic drainage, these nanoparticles that are delivered to the interstitium of the tumor are retained by a phenomenon known as enhanced permeation and retention (EPR) effect [25].

## RESULTS

### Patient characteristics

Sixteen patients signed consent and were enrolled and received at least one dose of study drug from 05/18/2009 to 02/01/2011. Patient characteristics are depicted in Table 1. All patients had received prior systemic chemotherapy and/or hormonal therapy. Two patients were removed during cycle 1 due to disease progression, and 1 patient was removed during cycle 4 due to noncompliance. All other patients completed 6 planned cycles. The tumor types enrolled are also shown in Table 1. The most common tumor types included ovarian and lung cancers.

**Table 1:** Patient characteristics

Characteristics	Number of patients
Total	16
Assessable for toxicity	16
***Age (years)***	
Median	62
Range	21-83
***Sex* (%)**	
Male	3 (18.75)
Female	13 (81.25)
***Ethnicity* (%)**	
White	10 (62.5)
Black or African-American	5 (31.25)
Hispanic	1 (6.25)
***Performance status* (%)**	
ECOG 0	7 (43.75)
ECOG 1	8 (50)
ECOG 2	1 (6.25)
***Tumor types* (%)**	
Ovarian	6 (37.5)
Endometrial	1 (6.25)
Lung	2 (12.5)
Breast	1 (6.25)
Non-Hodgkins Lymphoma	1 (6.25)
SLL/CLL	1 (6.25)
Sarcoma	1 (6.25)
Pancreatic	1 (6.25)
Biliary tract	1 (6.25)
Bladder	1 (6.25)

### Adverse events assessment- maximum tolerated does

All patients were evaluable for toxicities. In the group of the first 5 patients, one patient had grade 4 neutropenia for more than 8 days and three patients requiring dose reduction of nab-paclitaxel. These patients had had 4 or more previous lines of therapy. The eligibility criteria was modified with protocol amendment and enrollment was limited to patients with 2 or fewer prior lines of therapy. Ten patients completed all 6 cycles on this trial, one patient went off trial because of progression after 1 cycle and one patient was off trial after 3 cycles for PD, three patients were off trial because of adverse events (two for neutropenia and one for anemia) and one patient was off trial because of noncompliance. All treatment related toxicities are listed in Table 2.

**Table 2:** Treatment related toxicities

Toxicity	Grade 1-2 (%)	Grade 3 (%)	Grade 4 (%)	Grade 1-2 (%)	Grade 3 (%)	Grade 4 (%)	Grade 1-2 (%)	Grade 3 (%)	Grade 4 (%)
	Dose level 1 (75mg/m^2^) for patients 1-5			Dose level 2 (75mg/m^2^) for patients 6-9, 12, 13			Dose level 3 (100mg/m^2^) for patients 10, 11, 14-16		
**Non-hematological toxicities**									
Anorexia	0	0	0	16.7	0	0	20	0	0
Nausea	40	0	0	33.3	0	0	40	0	0
Vomiting	0	0	0	33.3	0	0	40	0	0
Dizziness	40	0	0	0	0	0	20	0	0
Dyspnea	20	0	0	0	0	0	0	0	0
Edema	20	0	0	16.7	0	0	0	0	0
Fatigue	60	0	0	66.7	0	0	60	0	0
Hot Flashes	0	0	0	16.7	0	0	0	0	0
Muscle weakness	40	0	0	16.7	0	0	40	0	0
Mucositis	20	0	0	16.7	0	0	0	0	0
Neuropathy	20	0	0	88.3	0	0	20	0	0
Puritis	20	0	0	17	0	0	0	0	0
Rash	20	0	0	0	0	0	0	0	0
Shortness of Breath	20	0	0	0	0	0	0	0	0
Weakness	40	0	0	16.7	0	0	40	0	0
**Hematological toxicities**									
Anemia	40	40	0	33.3	0	0	0	0	0
Leukopenia	20	40	20	0	33.3	0	0	20	40
Neutropenia	0	20	60	0	33.3	0	0	0	60
Platelets	20	0	0	0	0	0	20	0	0

### Response

Thirteen patients out of sixteen were evaluable for response criteria having completed at least 3 cycles of drug and received a scan. Tumor response is summarized in Table 3. One patient was off trial after one cycle because of rapid progression of disease. Eight out of thirteen patients, 61.5% (95% CI, 35%-87.95%), had an objective response. The clinical benefit rate [CR, PR and stable disease (SD)] of 92.3% (95% CI, 77.8%-106.8%) was observed. Three out of thirteen patients, 23% (95% CI, 0.12%-45.88%), had CR while 38.4% (95% CI, 11.96%-64.84%) had PR. Clinical activity by RECIST criteria included 1 complete response (CR) in refractory DLBC lymphoma, 2 CR in ovarian cancer by CA125, 4 partial responses (PR) in ovarian and endometrial cancer, 4 stable diseases (SD) in lung, sarcoma and pancreatic cancer, 1 unconfirmed PR in breast cancer, and 1 progression of disease in CLL/SLL.

**Table 3:** Tumor Response [n = 16, 10 subjects evaluable for response having completed at least 3 cycles]

Patient #	Cancer type	No. prior cytotoxic therapies	Cycles completed	Status	Best response
01	DLBC NHL	2	6 cycles	Completed	CR
02	Ovarian	5	6 cycles	Completed	PR
03	Ovarian	6	6 cycles	Completed	CR
04	Ovarian	6	1 Cycle	Neutropenia	N/A
05	Uterine	1	6 cycles	Completed	PR
06	NSCLC	2	6 cycles	Completed	SD
07	Ovarian	1	5 Cycles	Chemo induced anemia	PR
08	Ovarian	2	6 cycles	Completed	PR
09	Pancreatic	1	6 cycles	Completed	SD
10	Biliary tract	1	1 Cycle	PD	N/A
11	Sarcoma	1	4 Cycles	Non compliance	SD
12	SLL/CLL	2	3 Cycles	PD	PD
13	Breast	6	6 cycles	Completed	PR
14	NSCLC	1	6 cycles	Completed	SD
15	Bladder	2	1 Cycle	Neutropenia	N/A
16	Ovarian	2	6 cycles	Completed	CR

### Biomarker

Serum SPARC levels were correlated for clinical response and no correlation was observed.

## DISCUSSION

This phase I dose escalation study was designed to determine the toxicities and clinical response of the combination of two FDA approved and well-tolerated agents. The trial was to determine the maximum tolerated dose and preliminary efficacy for the combination. This trial of 16 patients with advanced cancers found that the MTD combination of 75mg/m^2^ of azacytidine daily for 5 days, followed by 100mg/m^2^ of weekly nab-paclitaxel was well tolerated in pretreated patients with diverse cancer types. At this dose level, no grade 4 toxicities were seen. Grade 3 toxicity consisted mostly of neutropenia

It is important to note that eight out of thirteen patients (61.5%) who were evaluable had an objective response. Remarkably, complete responses were seen with this combination in refractory cases of diffuse large B-cell lymphoma and ovarian cancer. Partial responses were seen in a wide range of cancers. Interestingly, all seven patients with women's cancer, i.e. breast, ovarian and endometrial cancers, who were evaluable for response experienced an objective response

The choice of using azacytidine priming was to increase SPARC levels within the tumor, which may enhance accumulation of albumin-bound drugs at the tumor site. Increased levels of SPARC is responsible for the enhanced transport of albumin and as a consequence increase sensitivity to nab-paclitaxel is an attractive strategy

In a recent study, azacytidine priming followed by standard doxorubicin-based combination chemotherapy was found to yield high rate of complete response in patients with high risk DLBCL. This was found to be correlated with increased expression of SMAD1, a signal transducer protein involved in multiple signaling pathways, by the DNA methylating agent azacytidine, leading to the sensitization of lymphoma cells to genotoxic effect of doxorubicin [26]. The patient with refractory DLBCL in our study had a CR, which was achieved most likely through a different mechanism since nab-paclitaxel is not considered genotoxic. As discussed previously, the mechanism in this case may be the overexpression of SPARC with resultant accumulation of nab-paclitaxel at tumor site. However there may be other as yet unknown mechanisms involved

We performed SPARC expression analysis in serum obtained at baseline, during and after therapy, but did not discern any correlation with response. Tissue SPARC expression might have been a better approach to evaluate correlation of changes in SPARC expression with treatment and clinical responses; however, it was not performed since it would not be practical to repeatedly biopsy metastases in these patients during treatment.

## MATERIALS AND METHODS

### Treatment plan

This was a phase I, open-label, staggered, sequential dose escalation study to determine the maximal tolerated dose (MTD) and overall safety profile of azacytidine when given with nab-paclitaxel for patients with advanced solid tumors. Patients in this phase I part of the study were enrolled at the Michell Cancer Institute, University of South Alabama, Mobile, Alabama and Huntsman Cancer Institute, University of Utah, Salt Lake City, Utah. The protocol was approved by the Institutional Review Board at the University of South Alabama, and University of Utah. It was conducted in accordance with the ethical principles originating from the Declaration of Helsinki and with Good Clinical Practice as defined by the International Conference on Harmonization. All patients gave written informed consent before enrollment. The trial was registered with http://www.ClinicalTrials.gov with the title A Phase I/II Clinical Trial of Vidaza with Abraxane in the Treatment of Patients with Advanced or Metastatic Solid Tumors and Breast Cancer (VA) and assigned identifier NCT00748553.

### Eligibility criteria

Inclusion criteria for this phase I trial was any solid tumor, including lymphoma that had progressed on at least one prior therapy in the recurrent or metastatic setting. The criteria was subsequently modified after the first three patients, to no more than two prior therapies. Other criteria included an eastern cooperative oncology group (ECOG) performance status of ≤ 2, adequate hematological parameters, kidney and liver function. Patients agreed to use appropriate methods of contraception while on study medication during and up to three months post last treatment and were able to give a written informed consent. Exclusion criteria include any surgery, radiotherapy or chemotherapy within 4 weeks of day 1 of treatment, known brain metastasis, prior use of taxanes with in the past 6 months, active infection requiring treatment, grade 2 or greater motor or sensory neuropathy, known or suspected hypersensitivity to azacitidine or mannitol, pregnancy, breast feeding or any other condition in the investigator's opinion that the patient was not eligible.

### Study design and treatment

The standard 3+3 design was used for the dose escalation phase. Patients were accrued to each dose level in cohorts of up to 3-6 patients. Escalation continued until a dose limiting toxicity (DLT) was observed or the highest dose-level was reached. Patients were enrolled in cohorts of three and no intra-patient dose escalation was allowed. The DLT period was defined as the first four weeks of treatment. If two or more DLTs were observed then the maximal tolerated dose was been exceeded. If none of the 3 subjects experience a DLT then subsequent patients are enrolled into the next higher dose level. If one patient had a DLT then that dose cohort was expanded to six subjects. If only one of the six patients had a DLT then subsequent patients will be enrolled at the next higher dose level. If two of three or six patients had a DLT then the MTD had been exceeded and the lower dose level would be evaluated to define the MTD. Dose reductions were required for any patient with a DLT assessed after the DLT period of four weeks or at the discretion of the investigator if he/she felt the reduction was in the patient's best interest.

Dose-limiting toxicities (DLTs) are defined as grade 3 or higher nonhematologic toxicities (except nausea/vomiting and diarrhea unless this occurs despite maximal supportive care), grade 3 thrombocytopenia for more than 7 days, and any grade 4 hematologic toxicity, with the exception of asymptomatic grade 4 neutropenia or leukopenia for less than 8 days in the first cycle. If a patient did not complete one cycle of therapy, for reasons other than a DLT, a replacement subject was added to the same cohort level.

The study was to evaluate three dose levels of azacitidine [dose level −1: 50 mg/m2, dose level 1: 75 mg/m2, or dose level 2: 100 mg/m2, subcutaneously (SC) or intravenously (IV)] with fixed dose of nab-paclitaxel (100 mg/m2 IV weekly). For each cycle, azacitidine will be given daily x 5 days, Monday through Friday (Days 1-5), and nab-paclitaxel will be administered the following Monday (Day 8) weekly times three weeks. Each cycle will be repeated every 4 weeks.

The baseline evaluation included a physical exam, ECOG performance status, tumor measurements, clinical staging, laboratory tests (complete blood count with differential, serum chemistry, and liver function tests), and serum pregnancy test. On day 1 of each cycle the patient had a physical exam, performance status, and laboratory evaluations. During cycle 1 on days 8, 15, and 22 laboratory evaluations and toxicity assessments were performed. In cycle two and beyond the laboratory evaluations and toxicity assessments were performed only on days 1 and 15. Adverse events were graded using the National Cancer Institute Common Terminology Criteria for Adverse Events, Version 3.0.

Tumor assessment and serum markers (if applicable) were assessed after every two cycles and at the completion of the study. Disease response and progression were evaluated by RECIST v1 criteria.

### Dose modification and treatment guidelines

Dose reductions of nab-paclitaxel were allowed for any patient who developed a neutropenic fever, had treatment delayed for more than 1 week or omitted due to low blood counts, had grade 3 or 4 thrombocytopenia, or had grade 2 or higher peripheral neuropathy. Dose reductions to 80mg/m^2^ and 60mg/m^2^ were allowed.

The first nab-paclitaxel dose of each cycle was delayed if the absolute neutrophil count was less than 1200/μl or the platelet count was less than 100,000/μl. The second and third nab-paclitaxel infusions were omitted if the absolute neutrophil count was less than 1000/μl or if the platelet count was less than 75,000/μl.

Azacytidine was delayed if the white blood cell count was less than 3000/μl, the absolute neutrophil count was less than 1500/μl, or the platelet count was less than 75,000/μl. Azacytidine dose was modified based on nadir blood counts.

Subjects were removed from study if a serious adverse event at the judgment of the investigator, lack of therapeutic effect, withdrawal of consent, lost to follow-up, protocol violation, or patient death.

### Statistical analysis

Subjects who received study medication were included in the safety analysis. Safety data included adverse events, laboratory data, vital signs, and physical exam findings. The MTD was determined based on 6 patients. Thus, for each MTD, common toxicities (occurring in ≥30% of patients) would rarely be unobserved (*P* = 0.11), and very common toxicities (occurring in 50% of patients) would almost never be missed. The best response, including complete response (CR), partial response (PR), stable disease (SD), or progressive disease (PD), for each patient was summarized. Descriptive statistics was used to summarize all patient characteristics, treatment administration, and compliance.
